# Case Report: Severe Complement-Mediated Thrombotic Microangiopathy in IgG4-Related Disease Secondary to Anti-Factor H IgG4 Autoantibodies

**DOI:** 10.3389/fimmu.2020.604759

**Published:** 2021-02-11

**Authors:** Gautier Breville, Ido Zamberg, Salima Sadallah, Caroline Stephan, Belen Ponte, Jörg D. Seebach

**Affiliations:** ^1^Department of Medicine, Division of Immunology and Allergy, Geneva University Hospitals, Geneva, Switzerland; ^2^Department of Clinical Neurosciences, Division of Neurology, Geneva University Hospitals, Geneva, Switzerland; ^3^Department of Medicine, Division of General Internal Medicine, Geneva University Hospitals, Geneva, Switzerland; ^4^Department of Anaesthesiology, Division of Anaesthesiology, Clinical Pharmacology, Intensive Care and Emergency Medicine, Geneva University Hospitals, Geneva, Switzerland; ^5^Département de médecine de laboratoire et pathologie, Service d’immunologie et d’allergie, Centre Hospitalier Universitaire Vaudois, Lausanne, Switzerland; ^6^Department of Medicine, Immuno-Hematology Unit, Geneva University Hospitals, Geneva, Switzerland; ^7^Department of Medicine, Division of Nephrology, Geneva University Hospitals, Geneva, Switzerland

**Keywords:** thrombotic microangiopathy, atypical hemolytic uremic syndrome, IgG4-related disease, antibodies, complement factor H-related protein, complement factor H, anti-factor H auto-antibodies, SARS CoV2

## Abstract

**Objective:**

To first describe and estimate the potential pathogenic role of Ig4 autoantibodies in complement-mediated thrombotic microangiopathy (TMA) in a patient with IgG4-related disease (IgG4-RD).

**Methods:**

This study is a case report presenting a retrospective review of the patient’s medical chart. Plasma complement C3 and C4 levels, immunoglobulin isotypes and subclasses were determined by nephelometry, the complement pathways’ activity (CH50, AP50, MBL) using WIESLAB^®^ Complement System assays. Human complement factor H levels, anti-complement factor H auto-antibodies were analyzed by ELISA, using HRP-labeled secondary antibodies specific for human IgG, IgG4, and IgA, respectively. Genetic analyses were performed by exome sequencing of 14 gens implicated in complement disorders, as well as multiplex ligation-dependent probe amplification looking specifically for *CFH, CFHR1-2-3, and 5*.

**Results:**

Our brief report presents the first case of IgG4-RD with complement-mediated TMA originating from both pathogenic *CFHR* 1 and *CFHR* 4 genes deletions, and inhibitory anti-complement factor H autoantibodies of the IgG4 subclass. Remission was achieved with plasmaphereses, corticosteroids, and cyclophosphamide. Following remission, the patient was diagnosed with lymphocytic meningitis and SARS-CoV-2 pneumonia with an uneventful recovery.

**Conclusion:**

IgG4-RD can be associated with pathogenic IgG4 autoantibodies. Genetic predisposition such as *CFHR*1 and *CFHR*4 gene deletions enhance the susceptibility to the formation of inhibitory anti-Factor H IgG4 antibodies.

## Introduction

IgG4-related disease (IgG4-RD) is a protean fibro-inflammatory condition characterized by tumefactive and hyperplastic lesions with storiform fibrosis and dense lymphoplasmocytic infiltrates rich in IgG4-positive plasma cells (1). Depending on the referenced cut-off level, elevated serum IgG4 concentrations can be detected in up to 80% of the cases, but are not exclusive to IgG4-RD being also present in a broad spectrum of other autoimmune diseases, allergic conditions, neoplasia, and Castleman’s disease (2). Histopathological analysis of biopsy specimens remains, therefore, the cornerstone of diagnosis of IgG4-RD. Current diagnostic criteria are based on organ involvement (dysfunction, localized, or diffuse swelling), serum IgG4 concentration (> 1.35 g/l), number of IgG4-positive plasma cells in tissue (>10 IgG4+ cells per high-power field) or the ratio of IgG4 to IgG (3). In comparison, IgG4 is the rarest of all four IgG subclasses, representing only 3–6% of total IgG; despite sharing more than 95% homology, differences in the amino-acid sequence in the second constant domain lead to weak or negligible binding to both C1q (classical complement pathway activation) and Fcγ receptors (4). Thus, IgG4 is believed to have mainly neutralizing and anti-inflammatory functions due to limited complement fixation and crosslinking, although this remains controversial (1).

Thrombotic microangiopathy (TMA), also known as atypical hemolytic uremic syndrome, is characterized by hemolytic anemia, thrombocytopenia, and organ failure (often renal) related to vascular damage provoking arteriolar and capillary thrombosis (5, 6). Complement-mediated TMA, an urgent life-threatening syndrome, results from uncontrolled activation of the alternative pathway of complement. In most cases, a hereditary origin can be identified, related to genetic abnormalities such as single nucleotide polymorphisms in the genes of complement factor H (CFH) and CD46, copy-number variations in the CFH-related 1 and 3 genes (CFHR), and fusion or deletion of genes in the CFHR region caused by non-allelic homologous recombination (5). In addition, mutations that can lead to lower activity levels of other complement inhibitor factors such as MCP and Factor I have been described. In contrast, 6-10% of complement-mediated TMA are acquired and linked to the presence of inhibitory autoantibodies directed against CFH (7).

## Case Report

**Figure 1:**
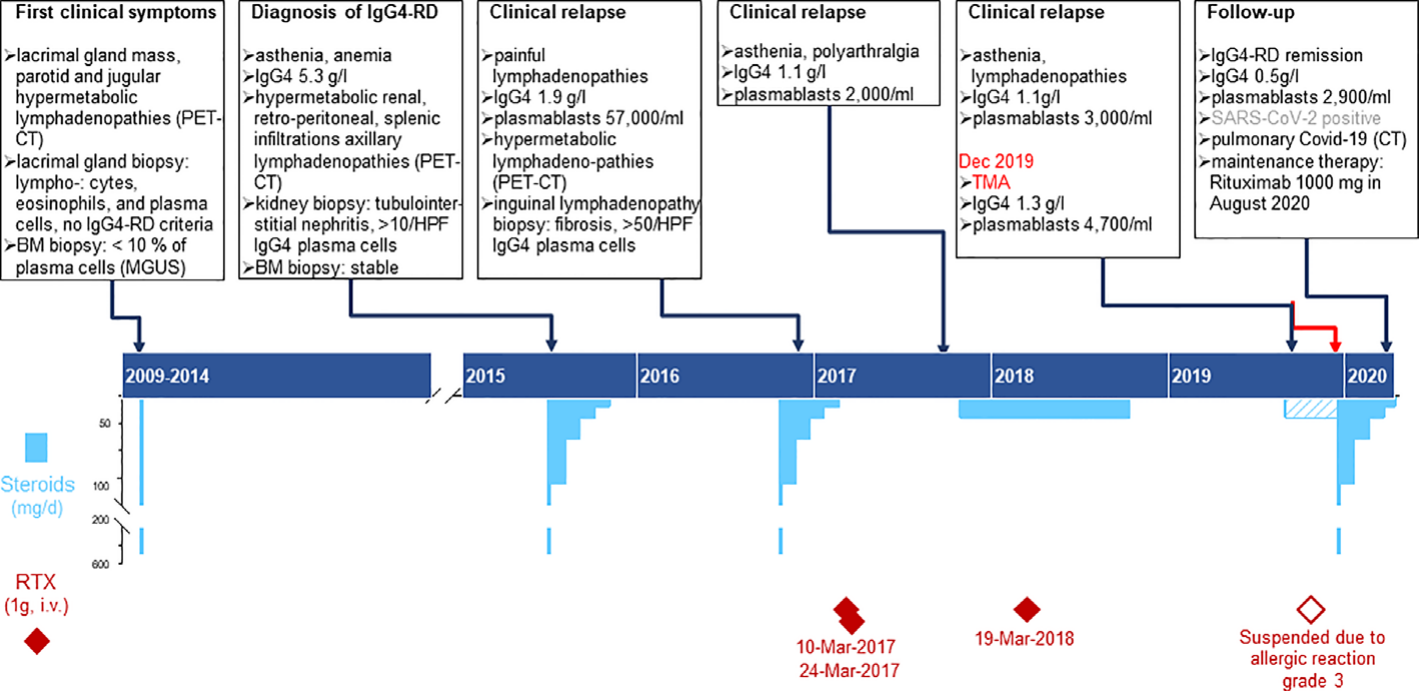
Time course depicting the clinical presentation of IgG4RD, laboratory results and treatment plans from 2009 to 2020. Chronic phase of the patient’s disease: the upper part summarizes the specific features of clinical findings, biopsy, imaging, and immunological laboratory results; the lower part shows the therapy, specifying dose, duration, and dates for Rituximab infusions (deep red diamonds) and steroids (sky blue bars). Pulse treatment with methyl-prednisone (500mg i.v.) is represented by the bottom scale blue bars and was usually followed by oral Prednisone (top scale blue bars); the dashed bar corresponds to the period when the patient did not adhere thoroughly to the prescribed therapy.

A 45-year-old woman, born in Cameroon, presented in December 2019 at the Emergency Department (ED) with a three-day history of epigastric abdominal pain, nausea, vomiting, and macroscopic hematuria. The patient had a clinical history of IgG4-RD diagnosed in 2015 with elevated serum IgG4 (5.3 g/l), plasmablast counts (57,000/ml), and tubulointerstitial nephritis with more than 10/HPF IgG4+ plasma cells. She was treated with corticosteroids and rituximab but had multiple relapses, as summarized in **Figure 1**. Other comorbidities included IgA lambda monoclonal gammopathy of undetermined significance (MGUS), α-thalassemia minor and a pituitary microadenoma with hyperprolactinemia. Four months before ED consultation, the patient had complained of worsening fatigue associated with cervical and submaxillary swellings. Flow cytometry revealed increased peripheral blood plasmablast counts (3,010/ml, normal: < 900), while the serum IgG4 level was normal (1.1 g/l, **Figure 1**). A relapse of IgG4-RD was suspected and treatment with oral prednisone 20 mg/d initiated with partial clinical regression despite an incomplete adherence to the treatment. An infusion of rituximab (1,000 mg) was prescribed but had to be discontinued due to a grade III anaphylactic reaction.

**Figure 2:**
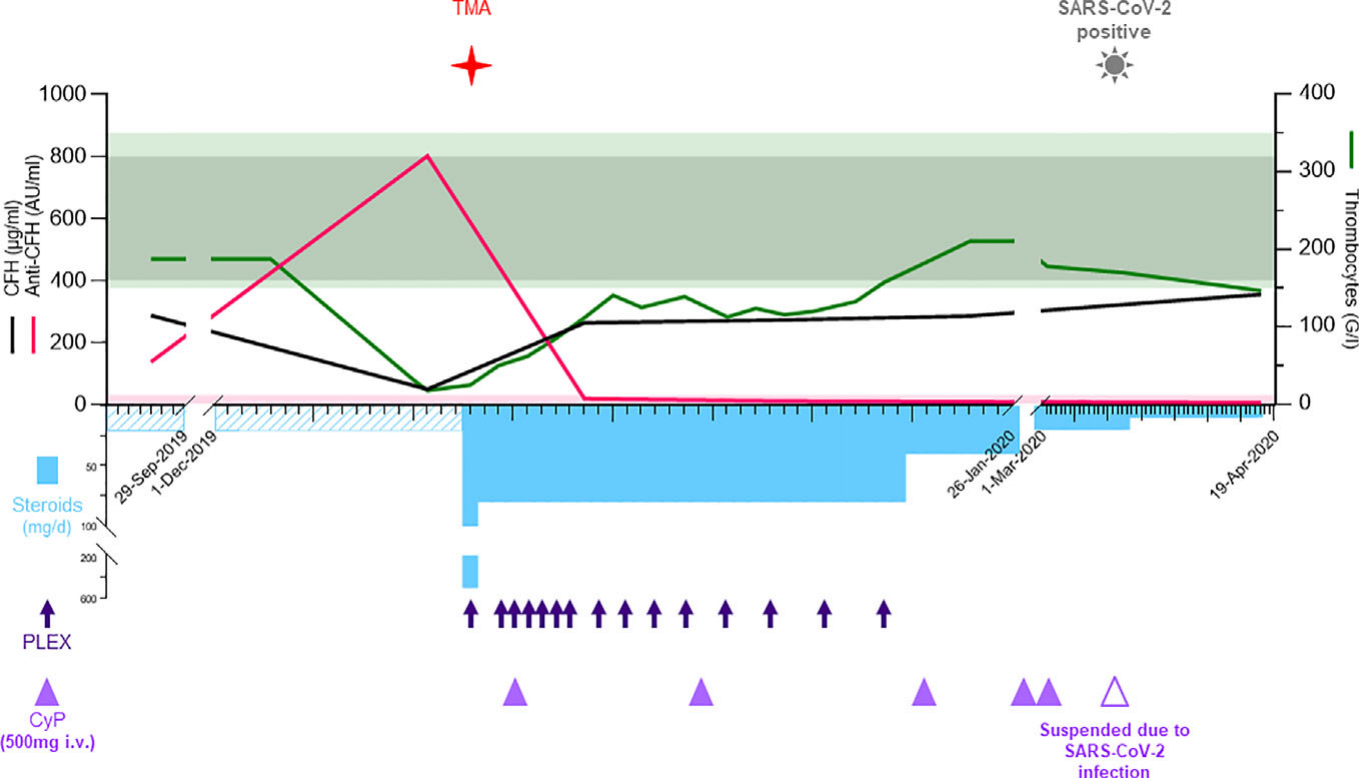
In the acute phase, the upper part shows the laboratory values for CFH (black, μg/ml), anti-CFH (pink, AU/ml), left y-axis, and thrombocytes (green, G/l) at the right y-axis. The normal reference values are shown with shades matching the colors of the different parameters. The lower part depicts the therapy including methylprednisolone/prednisone (sky blue bars, mg/d), plasma exchanges (deep blue arrows), and cyclophosphamide (purple triangles, 500 mg i.v.). The time when TMA and COVID-19 were diagnosed are shown with symbols. AU, arbitrary units; BM, bone marrow; Covid-19; coronavirus disease 2019; CT, computed tomography; CTX, cyclophosphamide; HPF, high-power field; IgA, immunoglobulin A; IgG4-RD, immunoglobulin G4-related disease; i.v., intra venous; PET-CT, positron emission tomography-computed tomography; PLEX, plasma exchange; RTX, Rituximab; SARS-CoV-2 severe acute respiratory syndrome coronavirus 2; TMA, thrombotic microangiopathy.

On admission, blood tests revealed hemolytic anemia (hemoglobin 100 g/l) with undetectable haptoglobin, increased LDH levels at 992 U/l, 2% of schistocytes on the blood smear, thrombocytopenia (35 G/l), and acute kidney injury with creatinine measured at 369 µmol/l for a baseline value of 77 µmol/l (KDIGO stage 3). Leucocyturia, glomerular hematuria, and nephrotic range glomerular proteinuria (estimated over 6.6 g/day) were present. Blood albumin levels were 28 g/l. Specific coagulation parameters (TP, PTT, and fibrinogen) were in the normal range. ADAMTS13 (a disintegrin and metalloproteinase with a thrombospondin type 1 motif, member 13) activity was normal (78%), and antiphospholipid antibodies were absent. Antinuclear antibodies (ANA) titers were at 1/5,000 with homogenous and nucleolar pattern without anti-nucleoprotein or -nucleosome specificities. Total IgG and IgG4 levels were at normal range, 11.1 and 1.2 g/l, respectively. Plasma complement C3 was significantly decreased, 0.14 g/l (0.66–1.35), with normal plasma C4 levels 0.16 g/l (0.08–0.34); the results of the last control two years earlier were 1.19 and 0.21 g/l, respectively. Activity of all three complement pathways, classical, alternative and lectin, was decreased: CH50 25% (Normal: 70–140), AP50 0% (Normal > 71), MBL 27% (Normal > 49). Detectable complement factor H (CFH) levels were low at 49 µg/ml (400–800, **Figure 2**) with high levels of anti-CFH antibodies detected at 801 AU/ml (Normal < 30, **Figure 2**). Intriguingly, elevated levels of anti-CHF antibodies and decreased FH activity were already present in frozen stored samples from 4 months before the clinical manifestation of TMA (**Figure 2**), but not from 1 year earlier (data not shown). Plasma complement C3 and C4 were determined by nephelometry while the determination of the complement pathways activity (CH50, AP50, MBL) were done using WIESLAB^®^ Complement System assay (SVAR life science AB.SE.) in the serum. Human Complement factor H levels were determined using ELISA-Hycult biotech. Com. in the plasma and anti-CFH antibodies were measured using ELISA-VIDITEST from VIDIA.CZ in the serum.

Both clinical and laboratory features were compatible with a diagnosis of complement-mediated TMA caused by inhibitory antibodies against CFH. While waiting for the results of CFH and anti-CFH antibodies levels, the patient was treated with daily plasma exchanges (PLEX, 13 cures in total) by fresh frozen plasma twice the plasma volume and corticosteroids (methylprednisolone 500 mg iv, followed by oral prednisone 1 mg/kg/day) with gradual tapering until May 2020 (**Figure 2**). Treatment with eculizumab was considered but not retained due to stabilization of laboratory parameters, including CFH and anti-CFH antibodies levels (263 µg/ml and 19 AU/ml, respectively). Withholding eculizumab was based on a joint decision between the medical staff and the patient after presenting the risks, benefits, and economic burden. However, the patient was vaccinated against meningococci and eculizumab was retained as a second line treatment option in case of non-response or premature relapse. To treat the underlying immunological condition iv cyclophosphamide was initiated one week later for a total of six infusions (500 mg each) over three months. Kidney function, proteinuria, hemolysis, thrombocytopenia, and complement activity progressively normalized. After five weeks of treatment, the patient achieved global clinical response and laboratory remission. In March 2020, the patient was diagnosed with lymphocytic meningitis and bilateral pneumonia due to SARS-CoV-2 infection with an uneventful recovery. Rituximab maintenance therapy (1,000 mg infusion) was administered in August 2020 using an induction of tolerance protocol and upon her last follow up in November 2020 the patient is free of symptoms with normal laboratory levels.

**Figure 3:**
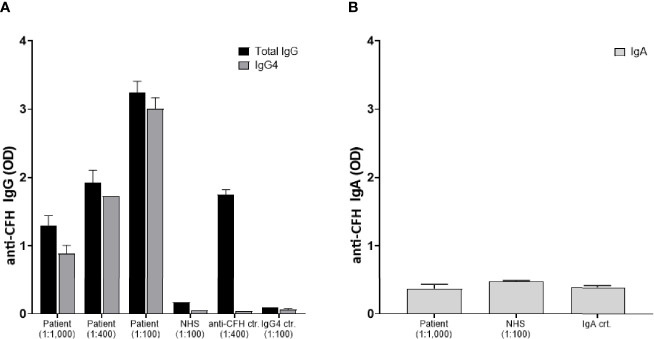
Identification of IgG4 anti-factor H autoantibodies: Plasma samples were analyzed for anti-factor H antibodies by a previously reported specific ELISA which was developed using Horse Radish peroxidase (HRP)-labeled secondary antibodies specific for human IgG, IgG4, and IgA, respectively (8). Data are shown as optic density at 490 nm (OD). **(A)** The patient’s serum was tested in duplicates at different dilutions 1:100, 1:400, and 1:1,000 for both IgG (black bars) and IgG4 (grey bars). Controls included a normal human serum (NHS), a serum with known elevated anti-CFH IgG levels, and a serum with elevated IgG4 but no anti-CFH activity. **(B)** Absence of IgA anti-factor H autoantibodies: Specific anti-CFH IgA antibodies were measured in the patient’s serum (total IgA level of 6.4 g/l), NHS, and another serum with a high total IgA concentration of 12.4 g/l.

In the context of IgG4-RD we further elucidated whether the anti-factor H inhibitory antibody belonged to the IgG4 subclass. Results are presented in **Figure 3A** demonstrating a significant anti-CFH total IgG elevation mainly of IgG4 subclass. Of note, the total serum IgG4 level was 1.2 g/l at that time. In the light of the known MGUS of the patient with IgA lambda paraproteinemia (6.4 g/l) and a previously published case (9), specific anti-CFH IgA antibodies were measured. Results indicate an absence of specific anti-CFH IgA (**Figure 3B**). Finally, genetic analyses were performed by sequencing the exome of 14 gens implicated in complement disorders (Twist Human Core Exome+RefSer_V1 EF Multiplex, Illumina NextSeq500), as well as multiplex ligation-dependent probe amplification looking specifically for *CFH*, *CFHR1-2-3* and *5* (kit MRC Holland SALSA p236_A3 + kit custom by V Fremeaux-BAcchi, Paris). Deletions in the genes of CFHR 1 and *CFHR 4*, GRCh37/hg19 chr.1:g.(196789032_196794607)_(196887536_196913011)del, were identified that are known risk factors for complement-mediated TMA (10).

## Discussion

To our knowledge, we present here the first case of complement-mediated TMA secondary to deletions in the *CFHR 1* and *CFHR 4* genes associated with pathogenic IgG4-type anti-CFH antibodies in a patient suffering from IgG4-RD with major salivary gland enlargement, orbital disease, lymphadenopathy, and IgG4 nephritis.

IgG4-RD physiopathology is still not well elucidated. Nevertheless, effectors mechanisms may include B and T cell interactions, with a pathogenic role of CD4+ cytotoxic T lymphocytes (CTL) balanced by B-lymphocytes, as well as T helper lymphocytes (Th-2) and regulatory T cells (Treg) that also regulate B-cell differentiation and TGF-β-mediated tissue fibrosis. IgG4 overexpression and accumulation could be inflammatory leftovers of chronic inflammation that results from massive plasma cell production. IgG4 antibodies are supposed to be nonpathogenic in IgG4-RD (1). However, tissue IgG4 accumulation could modulate local inflammatory responses. Tissue fibrosis evolution may be the product of this accumulation in association with CD4+ CTL and Treg cytokines production (11).

On the other hand, pathogenic autoreactive IgG4 antibodies have been observed in other autoimmune diseases such as myasthenia gravis, directed against muscle-specific tyrosine kinase (MuSK) receptor subtype, pemphigus vulgaris, directed against desmoglein 1, and idiopathic membranous glomerulonephritis, directed against M-type phospholipase A2 receptor (1). Moreover, in patients with acquired thrombotic thrombocytopenic purpura (TTP) related to anti-ADAMTS13 autoantibodies, specific IgG subclasses have all been detected but with a clear predominance of IgG4 (12, 13). Interestingly, IgG4 pathogenic autoantibodies directed against ADAMTS13 were reported in a patient with IgG4-RD causing acquired TTP (14). Our observation illustrates the potential pathogenic role of IgG4 and adds complement-mediated TMA to the list of diseases possibly caused by autoreactive IgG4 antibodies. Finally, since IgG4 polyclonal proteins have been reported incorrectly as M-protein bands in electrophoretic analysis (15) and the patient had longstanding stable IgA lambda MGUS, it was important to clearly distinguish IgG4 from IgA in our analysis. To this end, the concurrent presence of IgA MGUS and elevated IgG4 levels was confirmed by specific nephelometry and ELISA assays.

Complement-mediated TMA results from uncontrolled activation of the alternative pathway, and CFH autoantibodies are found in approximately 10% of reported cases. CFH is a major complement regulatory factor that acts as a cofactor for complement factor I (serine protease) converting C3b to an inactive form, as a decay-accelerating factor *via* competing with complement factor B in binding to C3b, and dissociates the alternative C3 convertase with formation of C3b and Bb. CFH-related protein 1 (CFHR1) is likely to inhibit the formation of C5 convertase and may compete with CFH for binding to C3b. Deficiency of CFHR1 can arise from homozygote *CFHR1/4* gene deletions (10). The majority of patients with CFH autoantibodies exhibited complete deficiency of CFHR1 and CFHR3 secondary to homozygous genes deletion (10). Our case revealed both acquired anti-CFH IgG4 autoantibodies and a genetic predisposition with *CFHR 1* and *CFHR 4* gene deletions. The later are considered as risk factors for complement-mediated TMA (10), since they may lead to structural changes that increase the antigenicity of CFH, or decrease the basal levels of effective CFHR (both in quantity and in function). CFHR alterations could therefore lead to a lower threshold for complement-mediated TMA in the presence of inhibitory anti-CFH IgG4 antibodies. Uncontrolled activation of the complement cascade triggers endothelial dysregulation, inflammatory reactions with leucocyte recruitment, and platelet activation resulting in tissue damage and thrombus formation, especially in the kidney (16, 17).

Therapeutic strategies for complement-mediated TMA are evolving with the recent introduction of complement inhibitors in particular eculizumab, a monoclonal antibody that binds complement component C5 and prevents its cleavage by C5 convertases and formation of the membrane attack complex (MAC) (18). In our case, eculizumab was considered early in the treatment strategy but was not administered due to a rapid and satisfactory clinical and biological response to plasma exchanges and corticosteroids. Our intention was to treat the underlying disease, ie IgG4-RD, in order to efficiently suppress further production of inhibitory anti-CFH IgG4 autoantibodies by induction with cyclophosphamide followed by maintenance treatment using rituximab.

Severe infections such as HIV, influenza, and pneumococcus are known causes of TMA and related to relapse (19). In our patient, COVID-19 infection was uneventful without any signs of clinical or biological relapse during a six-week follow up. According to the recent finding that severe COVID-19 infection is associated to systemic endotheliitis (20), the fact that our patient was immunosuppressed might have been a protective factor. Nevertheless, this hypothesis requires more research on the mechanisms of severe COVID-19 and the role of immunosuppression.

In conclusion, we describe for the first time a pathogenic role of IgG4 autoantibodies directed against CFH causing complement-mediated TMA in a patient with IgG4-RD and a genetic predisposition with homozygote *CFHR1/4* gene deletion.

## Ethics Statement

Ethical review and approval were not required for the study on human participants in accordance with the local legislation and institutional requirements. The patient provided their written informed consent to participate in this study.

## Author Contributions

Conceptualization and design of the study: GB, IZ, BP, and JS. Acquisition and analysis of data: GB, IZ, SS. Drafting of a significant portion of the manuscript or figures: GB, IZ. Correction of the manuscript: GB, IZ, CS, BP, JS. All authors contributed to the article and approved the submitted version.

## Conflict of Interest

The authors declare that the research was conducted in the absence of any commercial or financial relationships that could be construed as a potential conflict of interest.

## Abbreviations

CH50, haemolytic complement 50%; AP50, alternative pathway 50%; CFH, complement factor H; CFHR, CFH-related protein; IgG4-RD, IgG4-related disease; MGUS, monoclonal gammopathy of undetermined significance; MBL, Mannose Binding Lectin.
